# Asymptomatic *Leishmania donovani* infection and associated factors among blood donors attending at Metema district Blood Bank, Northwest Ethiopia: a cross- sectional study

**DOI:** 10.1186/s13690-023-01082-7

**Published:** 2023-04-21

**Authors:** Iyasu Melkie, Mulat Yimer, Getaneh Alemu, Banchamlak Tegegne

**Affiliations:** 1 Bahir Dar city administration Blood Bank, Bahir Dar city, Ethiopia; 2 Department of Medical Laboratory Science, College of Medicine and Health Sciences, Bahir Dar University, Bahir Dar city, Ethiopia; 3 Medical Parasitology Department, Amhara Public Health Institute, Bahir Dar city, Ethiopia

**Keywords:** Asymptomatic *Leishmania donovani* infection, Blood donors, rK39 test, Direct agglutination test, Ethiopia

## Abstract

**Background:**

Ethiopia is one of the top 10 countries in the world where 90% visceral leishmaniasis cases are reported. Metema-Humera lowlands are the most important foci in Ethiopia. Blood transfusion in visceral leishmaniasis endemic foci in Ethiopia does not consider screening of visceral leishmaniasis during blood donation. The aim of this study is therefore, was to assess asymptomatic *Leishmania donovani* infection and associated factors among blood donors attending at Metema district Blood Bank, Northwest Ethiopia.

**Methods:**

A Health facility based cross-sectional study was conducted at Metema Blood Bank from February to March 2020. A total of 205 blood donors were eligible and participated in this study. Structured questionnaire were used to collect data on socio-demographic characteristics and perceived risk factors associated with asymptomatic visceral leishmaniasis among blood donors. Blood donors were screened using both rK39 and direct agglutination tests based on the manufactures’ instructions. Data were analyzed using SPSS version 20.0. Chi-square test was used to assess associations of *Leishmania donovani* infection with predisposing factors. Associations were considered statstically significant on P-value < 0.05 at 95% confidence level.

**Results:**

Of the total 205 participants, 32(15.6%) were positive for asymptomatic *Leishmania donovani* infection at least by one of the diagnostic tests used. Eight (3.9%) and 30(14.6%) of the participants` were positive by the rK39 and direct agglutination tests, respectively. Six (2.9%) donors were tested positive by both diagnostic tests. Family history of visceral leishmaniasis (X²=11.334, P = 0.003) and having neighbors with history of visceral leishmaniasis (X²=5.923, P = 0.015) were significantly associated with asymptomatic *Leishmania donovani* infection among blood donors.

**Conclusions:**

The prevalence of asymptomatic *Leishmania donovani* infection was 15.6%. Asymptomatic visceral leishmaniasis was significantly associated with donors’ family and neighbors’ history of infection. Therefore, laboratory screening of blood donors for visceral leishmaniasis in endemic areas will be mandatory. Moreover, this study will give base line information for future study in the country.

## Background

Leishmaniases are a group of diseases caused by infection with protozoan parasites from the genus *Leishmania* [1]. It is transmitted by the bite of the female *Phlebotomus* species in the Old World and *Lutzomyia* species in the New World [2]. Depending on the infecting *Leishmania* species and/or the immune status of the host and some other factors, some of them are localized at the skin and resulted in cutaneous forms as localized cutaneous leishmaniasis (LCL), muco-cutaneous leishmaniasis (MCL) and diffuse cutaneous leishmaniasis (DCL). While others infect visceral organs and cause visceral forms [3].

According to the recent World Health Organization (WHO) report, Ethiopia is one of the ten countries that have ˃ 90% annual incidence of visceral leishmaniasis (VL) [4]. In addition, East Africa accounted for 45% of global VL cases reported to WHO in 2018 [5].

In Ethiopia, both VL and CL are growing health problems [6]. VL accounted with annual incidence cases of 2,500 to 4,000. While CL with 20,000 to 30,000 incidence cases [3]. In Amhara Region, VL has been reported in Libokemkem, East and West Bellessa, West Armachiho, Quara, Metema and west Armachiho districts [7, 8]. It is also noted that 60% of the VL cases of the country is found in Amhara Region and of whom, one-third are VL/HIV co-infected [9].

Determinants for the outcome of *L. donovani* complex infections are still debatable. After infection, it is not possible to predict when and who will develop VL from asymptomatic *L. donovani* infected individuals [10]. As a result, whether the infection remains asymptomatic or progresses towards VL probably results from a complex interaction between host, parasite, and vector-related factors [11, 12].

All individuals infected with *L. donovani* complex, do not lead to overt clinical disease and most of them may stay asymptomatic for 10–12 years [13, 14]. In some infected individuals, VL is a progressive disease and characterized by excessive parasite replication in liver, spleen and bone marrow [15, 16]. In Ethiopia, even if, the comprehensive asymptomatic VL prevalence has not been yet known at the country level, the ratio of asymptomatic to clinical VL cases was reported to be 5.6:1 [17].

Although asymptomatic infection represents 25–80% [18], current evidences using indirect and direct xenodiagnoses depicted asymptomatic individuals having no role in the transmission of VL [19, 20]. In contrary to this, asymptomatic individuals are considered to be the potential reservoirs of VL and play a major role in the transmission of the disease in endemic and non-endemic areas [13, 21]. One of such transmission is via blood transfusion because of survival and infectivity of parasites under blood bank storage conditions [22]. The fact that Ethiopia is one of the ten countries with high burden of VL, the national blood donor selection guideline didn’t include screening for asymptomatic VL [23]. Hence, laboratory screening for VL detection in blood banks is necessary to ensure the safety of the blood donation process and the health of recipients, especially in endemic areas [24].

On the other hand, there is no standard and approved laboratory diagnostic method(s) for screening, detecting and identifying individuals with asymptomatic cases of VL [25]. As a result, a combination of laboratory diagnostic tests should be used for diagnosing individuals who were exposed to *L. donovani* parasite and remained asymptomatic [26]. Hence, the most commonly used laboratory diagnostic methods for asymptomatic VL are: rK39, DAT and others [13]. Of these, rK39 and DAT are most widely used methods because of their field applicability [27].

In addition, different socio-demographic characteristics, occupation related habits and ecological conditions contribute for the transmission of VL. However, there are variations in predisposing factors at different localities as reviewed by Gadisa and colleagues [28]. Hence, identifying VL associated risk factors in local contexts are important for effective intervention. Therefore, the aim of this study was to assess the prevalence of asymptomatic *L. donovani* infection and associated factors among blood donors attending at Metema Blood Bank in Northwest Ethiopia.

## Methods

### Study design, period and area

A Health facility based cross-sectional study was conducted at Metema Blood Bank from February to March 2020. Metema is one of the districts in the Amhara Region and previously it was part of the North Gondar zone, and currently it is part of the West Gondar zone. This district has a total population of 110,252. Of whom, 58,748 and 51,504 are men and women, respectively [29]. The district has an altitude ranging from 550 to 1,600 m above sea level and a mean annual temperature ranging from 22 to 28 °C. The annual rainfall ranges from 400 to 600 mm [person. Comm]. The natural vegetation of this district is predominantly acacia trees with grasses grown under them, and most of the soil is clay soil. A lot of small- and large-scale private and government-owned farms are in the district. Because of these opportunities, many daily laborers travel each year from June to December from the highland, VL non-endemic areas to this endemic area for weeding and harvesting of sesame, cotton, and corn [30]. Metema General Hospital serves as a referral hospital for 14 health centers and daily laborers who come to the area for the weeding and harvesting seasons.

### Sample size determination and sampling technique

Using purposive sampling technique, a total of 600 blood donors were screened during the study period. Of those, 205 blood donors were passed the screening criteria of the Ethiopian national blood bank service (National Blood Bank Service of Ethiopia, 2017) and gave consent to participate in the study and eligible for VL screening.

### Data and blood sample collection and processing

Socio-demographic characteristics and associated risk factors were collected using structured questionnaire. After 450ml blood was drawn from the blood donor, the remaining blood in the tube section was used for screening of VL with similar fashion in screening for other infectious diseases based on National Blood Bank Service of Ethiopia guideline. rK39 test (DiaMed-IT LEISH-Bio-Rad Laboratories, Marnes-la-Coquette- France) was performed at Metema Blood Bank according to the manufacturer’s instructions. In the meantime, DBS using Whitman filter paper was collected, allowed to air dried, packed in plastic zipper bags with desiccants. Then, stored at -20^o^C until shipped to Amhara Public Health Institute, Bahir Dar- Ethiopia for DAT test (ITMA-DAT/VL; Prince Leopold Institute of Tropical Medicine, Antwerp, Belgium) following the manufacturer’s instructions.

### Data analysis

Data were entered and analysed using Statistical Package for the Social Sciences version 20. Chi-square and P-value were used for assessing general associations between VL infections with perceived predisposing factors. Associations between variables were considered statistically significant if P-value$<$0.05 at 95% confidence level.

## Results

### Socio-demographic characteristics of study participants

In this study, a total of 205 blood donors were participated. Of those, 128 (62.4%) and 77 (37.6%) were males and females, respectively. The majority 144 (70.2%), of participants` age ranged from 18 to 20 years-old. Finally, 204 (99.5%) participants have attended formal education (Table 1).

**Table 1:** Socio-demographic characteristics of study participants attending Metema Blood Bank service from February to March 2020 (n = 205)

Characteristics	Frequency N (%)	Positive result N (%)
Sex Male Female	128 (62.4)	20 (15.6)
	77 (37.6)	12 (15.6)
Age groups (in years) 18–20 21–25 > 25	144 (70.2)	21 (14.6)
	47 (22.9)	8 (17)
	14 (6.8)	3 (21.4)
Educational status Not formal education Primary school Secondary school	1 (0.5)	0
	2 (1)	0
	202 (98.5)	32 (100)
Residence Rural Urban	179 (87.3)	30 (16.8)
	26 (12.7)	2 (33.3)
Occupational status Students Merchants Government employee	183 (89.3)	26 (14.2)
	18 (8.8)	5 (27.7)
	4 (2)	1 (25)

### Prevalence of visceral leishmaniasis

Of the total 205 eligible blood donors, 30 (14.6%) were positive for DAT tests. The overall prevalence by either of the diagnostic tests was 32 (15.6%) (Table 2).

**Table 2:** Prevalence of visceral leishmaniasis by rK39 and DAT tests at Metema Blood Bank, from February to March 2020

		DAT		Total N (%)
		Positive	Negative	
rK39	Positive	6	2	8 (3.9)
	Negative	24	173	197 (96.1)
	Total N (%)	30 (14.6)	175 (85.4)	205 (100)

### Factors associated with asymptomatic visceral leishmaniasis

In this study, risk of being infected with *L. donovani* was similar in both male and female blood donors (15.6%). However, no statistically significant differences between *L. donovani* and gender were observed (X^2^ = 0.00, P = 1.00). Of the total of 32 VL positive study participants observed, 21(14.6%), 8(17%) and 3(21.4%) were ages of 18–20, 21–25, and > 25 years, respectively. But, risk of being infected with *L. donovani* was not statistically associated with age group of blood donors (X^2^ = 0.546, P = 0.761). From total VL positive study participants; 26(14.2%), 5 (27.7%) and 1(25%) were students, merchants and government employee, respectively. However, there was no statistically significance difference among occupational status (X²=2.564, P = 0.277). On the contrary, risk of being infected with *L. donovani* was statistically associated in both blood donors with family history (x^2^ = 11.334, P = 0.003) and neighbors’ VL history (X^2^ = 5.923, P = 0.015) (Table 3).

**Table 3:** Association of visceral leishmaniasis with independent variables among blood donors attending at Metema Blood Bank from February to March 2020 (n = 205)

Variables		Frequency N (%)	Positive N (%)	X²	P-value
Sex	Male	128 (62.4)	20 (15.6)	0.00	1.0
	Female	77(37.60	12 (15.6)		
Age	15–20	144 (70.3)	21 (14.6)		
	21–25	47 (22.9)	8 (17)	0.546	0.761
	> 25	14 (6.8)	3(21.4)		
Occupation	Student	183 (89.2)	26 (14.2)		
	Merchant	18 (8.8)	5 (27.7)	2.564	0.277
	Employee	4 (2)	1 (25)		
Indoor residual spraying	Yes	170 (82.9)	28 (16.4)	0.789	0.374
	No	35 (17.1)	4 (11.4)		
Bed net utilization habit	Yes	161(78.5)	27(16.8)	1.012	0.315
	No	44 (21.5)	5 (11.4)		
Family VL history	Yes	27(13.2)	10 (37)		
	No	174 (84.9)	21 (12)	11.334	0.003
	Not know	4(1.9)	1(25)		
Neighbors with VL history	Yes	54(26.3)	14(26)	5.923	0.015
	No	151(73.7)	18(11.9)		

## Discussion

In transfusion medicine, infectious diseases (bacterial, viral and protozoan) pathogens are known, or suspected to be transmitted through blood transfusion [31]. To the best of our knowledge, this study is the first report of *L. donovani* infection among blood donors in Ethiopia and the study area.

The current study depicted that the prevalence of asymptomatic VL among blood donors was (15.6%, 95%CI: 12.2–19.0). This was in agreement with previous study done in Brazil with 15.6% VL prevalence [20] and 13.4% positivity among blood donors in South Sudan [32]. This similarity might be due to high burden of VL in Brazil, South Sudan and Ethiopia [33]. However, the present result is higher than earlier findings of 0.3% in Bangladesh, despite Bangladesh is also among the six high burden VL countries [33, 34]. This variation might be due to the diagnostic techniques difference as the study in Bangladesh used only rK39, unlike use of both rK39 and DAT tests in the present study. Furthermore, prevalence of asymptomatic VL in the present study from Ethiopia was also higher compared to similar results of 6.4% in Istanbul [35], 3.8% in Iran [36], and 3.1% in Spain [37]. Variation might be due to difference in parasites (*L. infantum* vs. *L. donovani*) or low transmission of VL in these countries. Or it might be due to solely anthoponotic in Indian Sub-continent vs. East African transmission.

Both rk39 and DAT are being used in Ethiopia for the diagnosis of VL suspected cases [3]. However, there is no approved diagnostic method (s) for screening of asymptomatically infected individuals. In the present study, DAT has shown positive reaction in 14.6% participants compared to only 2.9% positives by rk39. Low detection rate of rK39 test might be due to low performance and geographical location dependent variation of rK39 result [27, 38]. DAT test result in the present study was higher than other study done 9.7% in northeastern Brazil [39]. The difference in seropositivity due to could be the difference in *Leishmania* parasites (*L. donovani* vs. *L. infantum*). Or it might be due to population differences (Ethiopian vs. Brazillian) and cause seropositivity difference.

In this study, among those blood donors, whose houses were sprayed with insecticide, 16.4% of them were seropositive. The possible explanation might be due to the fact that the insecticide was not sprayed properly or they might be bitten outside their home. Likewise, of the study participants who had bed nets, 16.8% of them were seropositive. This could be improper utilization of bed nets (utilize bed nets for other purposes like: utilize bed nets for carrying straw, corn and teff). Hence, more liable for sand flies exposure and hence infected and became seropositive.

In the present study, family history and neighbors with history of VL were significantly associated with risk of being infected with *L. donovani*. This was in line with other studies in Nepal [40]. This might be due to the increment of carbon dioxide released by the population and attract large numbers of sand flies and liable for exposure and became seropositive.

## Conclusions

In this study, the prevalence of asymptomatic VL among blood donors in the study area was 32 (15.6%). In addition, asymptomatic VL was significantly associated with family history of VL among family members and neighbor’s .DAT test showed more sensitivity compared to rK39 in identifying asymptomatic VL among blood donors. Therefore, VL screening of blood donors should be started among the major blood bank sites in the region. In parallel to this, further research shall be done to know the comprehensive prevalence of asymptomatic VL and associated risk factors among blood donors in Amhara Region.

## Data Availability

All data associated with this manuscript is available in request upon the corresponding author.

## List of abbreviations

VL: Visceral leishmaniasis
CL: Cutaneous leishmaniasis
DAT: Direct agglutination test
HIV: Human Immuno Deficiency Virus
